# Viral Hybrid Vectors for Somatic Integration - Are They the Better Solution?

**DOI:** 10.3390/v1031295

**Published:** 2009-12-15

**Authors:** Nadine Müther, Nadja Noske, Anja Ehrhardt

**Affiliations:** Max von Pettenkofer-Institut, Department of Virology, Ludwig-Maximilians-Universität Munich, Pettenkoferstr. 9A, 80336 Munich, Germany

**Keywords:** hybrid vector, somatic integration, adenovirus, retrovirus, adeno-associated virus, herpes simplex virus, targeted integration, Sleeping Beauty transposase, PhiC31 integrase, zinc-finger nuclease

## Abstract

The turbulent history of clinical trials in viral gene therapy has taught us important lessons about vector design and safety issues. Much effort was spent on analyzing genotoxicity after somatic integration of therapeutic DNA into the host genome. Based on these findings major improvements in vector design including the development of viral hybrid vectors for somatic integration have been achieved. This review provides a state-of-the-art overview of available hybrid vectors utilizing viruses for high transduction efficiencies in concert with various integration machineries for random and targeted integration patterns. It discusses advantages but also limitations of each vector system.

## 1. Introduction

The development of novel and safer vector tools for stable maintenance of therapeutic DNA and transgene products within a cell, especially in rapidly dividing cells (e.g., bone marrow derived cells), is of great interest to the research community. For instance therapies for these genetic diseases would benefit from such tools because they rely on stable production of the defective gene product and subsequent long-term phenotypic correction. Furthermore, these vectors would circumvent repeated vector administration necessary for life-long correction. Moreover, inconveniences for the patient due to repeated drug administration could be avoided.

Recombinant viral vectors commonly used for stable gene transfer can be divided by their ability to either integrate foreign DNA into the host genome or by their capability to be maintained as episomal, non-integrative viral vector genomes. However, until now none of the commonly used vector systems for maintenance of therapeutic DNA in dividing cells is optimal: (i) the development of episomally maintained DNA molecules, although without any risk of insertional mutagenesis, remains a challenging task. Limiting features are insufficient retention and replication of the episomal viral (e.g., adenovirus and adeno-associated virus [AAV]) or non-viral (plasmid replicon) DNA molecule during cell division, inefficient delivery of replicating DNA, and the involvement of potential toxic viral coding products which in many cases are prerequisites for DNA retention within the nucleus. (ii) Integrating viral vectors were tested in preclinical and clinical settings and gathered information revealed that integration site preferences may cause genotoxicity by changing the expression profile and properties of the transduced target cell due to insertional mutagenesis. Therefore, one obvious approach to improve stable gene transfer for therapeutic applications is improving target site selection of integrating viral vectors by specifying their integration site preferences. To accomplish this goal, one solution could be the generation of viral hybrid vectors. Viral hybrid vectors are defined as vectors which combine the high transduction rates of viruses with genetic elements for stable maintenance of therapeutic DNA.

**Figure 1.**
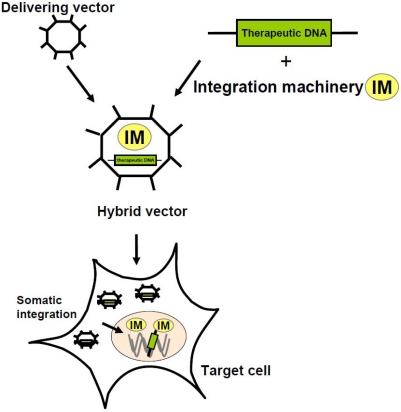
**Principle of integrating viral hybrid vectors.** Viral hybrid vectors combine efficient transduction rates of viruses (delivering vector) with improved genetic elements for stable maintenance of therapeutic DNA by somatic integration into the host genome of the target cell.

This article focuses on recombinant viral hybrid vectors with the ability to integrate their therapeutic DNA into the host genome of eukaryotic cells. The principle of integrating viral hybrid vectors is schematically shown in Figure 1. It is based on combining high efficiency gene transfer of viral vectors with the most advanced integration machineries. Herein, we describe the state-of-the-art of hybrid vector technologies for increased and safer integration of therapeutic DNA into the host genome and we discuss the advantages but also limitations for each single vector system.

The number of different hybrid vectors which are under development as gene-therapeutic integrating hybrid vectors is steadily increasing (Table 1. and 2.). Mainly, integrating viral hybrid vectors can be distinguished by their viral delivery vehicle and the utilized integration machinery. With respect to the delivery vehicle, previous studies have employed adenovirus, herpes simplex virus 1 (HSV-1), and retrovirus. Regarding the integration machinery, hybrid vectors can be subdivided into two groups according to how their genome integrates, by viral integration systems (AAV, retrovirus) or non-viral systems for integration (Sleeping Beauty [SB] transposase [1], bacteriophage integrase PhiC31 [2,3], and zinc-finger nucleases [ZFNs] [4,5]). All integration machineries are suitable tools for the generation of hybrid vectors because they allow stable and long-lasting transgene expression in dividing cells.

Integrating hybrid vectors are an attractive choice if stable alterations need to be maintained in dividing cells. Each of these different integrating viral hybrid vectors is characterized by a set of different properties coming from the delivering vector itself and the integration machinery which is used, that make it suitable for some applications in gene therapy and unsuitable for others. Herein, we classified the hybrid vector by its delivery vehicle (adenovirus, herpes simplex virus 1, retrovirus) and subdivided the respective group depending on the integration machinery employed (Table 1.).

## 2. Adenovirus-based hybrid vectors for somatic integration

Adenoviruses are non-enveloped viruses with an icosahedral capsid. The length of the linear, double-stranded adenoviral DNA genome is 26 to 45 kilobase (kb) in size [6]. Adenoviruses were isolated from various species and human adenoviruses belong to the genus *Mastadenoviridae*. Until now, 53 human adenoviral serotypes were identified which were divided into 6 subgroups (A–F). They display a very diverse and broad cellular tropism after *in vivo* application making them very attractive for gene transfer studies.

Recombinant adenoviral vectors (rAdVs) commonly used in gene transfer applications are usually replication-deficient and devoid of one or more essential genes. Based on the current knowledge, rAdVs predominantly persist as episomal, replication inactive vector molecules [7]. Integration efficiencies of rAdVs with approximately 10^−4^ to 10^−6^ integration events per infectious genome are rather low [8]. However, it is important to point out that in principle, adenovirus has the potential to integrate into the host genome of the infected cell. For instance human adenovirus serotype 12 integrates into the host genome and it shows transforming potential in rodents [9–11].

To date, recombinant adenoviral vector systems are the most efficient vectors for transferring genes into a large proportion of cells *in vitro* and *in vivo* [12]. Different recombinant adenoviral vectors have been developed with various regions of viral coding region removed [13]. Early first and second generation adenoviral vectors deleted for one (E1 gene) or several genes (E2a, E2b or E4) still result in cytotoxicity, because low-level transcription of viral genes from these adenoviral vectors served as potent antigene resulting in an effective host immune response [14–16]. The further development of helper-dependent adenoviral vectors (HDAdVs) significantly improved the toxicity and immunogenicity profiles of adenoviral vectors [14,17]. HDAdVs lack all viral coding sequences and the only adenoviral sequences left are the inverted terminal repeats (ITRs) and the packaging signal. The HDAdV production process splits the actual therapeutic gene to be transferred to one vector and the viral genes required for vector replication and production in a second helper virus vector whose packaging signal is flanked by Cre recombinase recognition (loxP) site [18]. In cells expressing the Cre protein, the packaging signal is excised from the helper virus genome and the therapeutic vector genome is preferentially packaged. In addition, due to the deletion of all viral genes, HDAdV has a high cloning capacity up to 37 kb. However, even for HDAdV there is some toxicity related to the incoming viral capsid proteins [19]. Duration of transgene expression after adenoviral gene transfers various. It depends on the promoter driving transgene expression [20], the delivery method [21], and the mouse strain and its immunological background [22], and at least in part on cell division and subsequent reduction of episomal vector genomes [23]. Therefore, the development of integrating adenoviral hybrid vectors would be one potential solution for achieving long-term transgene expression especially for cells with a high turnover.

**Figure 2.**
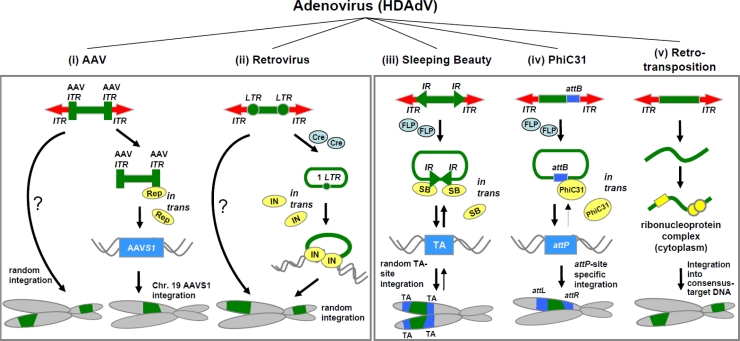
Schematic diagram of all integrating hybrid vector systems based on helper- dependent adenoviral vectors (HDAdV) as delivery vehicles (i–v). The transgene expression cassette (shown in green) and components for somatic integration are co-delivered using the recombinant adenoviral vector. Virus-based integration machineries are shown in the left panel and integration machineries based on non-viral systems are displayed in the right panel. (i) HDAdV/adeno-associated virus (AAV) hybrid vectors integrate into the host genome randomly via AAV internal terminal repeats (ITRs). Rep-Protein expressed *in trans* provided by a second vector mediates site-specific integration after binding to AAV-ITRs and to the AAV integration site S1 (AAVS1), located on the human chromosome 19. (ii) HDAdV/retrovirus (RV) hybrid vectors integrate transgene expression cassettes flanked by retroviral long terminal repeats (LTRs) directly or after circularization from the adenoviral vector genome mediated by Cre recombinase provided *in trans*. When the retroviral integrase (IN) is supplied *in trans*, the integration complex triggers the random insertion of the transgene into the host genome. (iii) For HDAdV/Sleeping Beauty (SB) transposase hybrid vectors the transgene is flanked by inverted repeats (IR) as a recognition site for the Sleeping Beauty (SB) transposase. SB is provided *in trans* together with Flp recombinase. When Flp mediates circularization of the DNA and therefore mobilization of the transposon from the HDAdV genome, SB binds to the recognition sites and integrates the transposon including the transgene randomly into the TA-sites of the host genome. This process is reversible. (iv) For the HDAdV/PhiC31 integrase hybrid vector system, the vector DNA harbours the PhiC31 recognition site *attB* within the therapeutic transgene. When PhiC31 protein is provided *in trans*, it mediates integration into a limited number of pseudo *attP* sites in the mammalian genome by unidirectional recombination. The circular form of the PhiC31 substrate is released from the adenoviral vector genome by Flp recombinase. At the time it is not clear whether this step is required. (v) Retrotransposition from the adenoviral vector genome is based on the interspersed element L1. After transduction of the target cell L1 and transgene is described and RNA leads to the generation of ribonucleoprotein structure in the cytoplasm. This complex is then imported into the nucleus for somatic integration into a consensus genomic target DNA.

The history of integrating adenoviral hybrid vectors for stabilized gene transfer is long and a variety of hybrid vectors have been emerging. They combine the highly efficient DNA delivery of adenoviral vectors with the integrating machinery of transposons (HDAdV/retrotransposon [24], HDAdV/Sleeping Beauty transposon [25]), phage integrases (HDAdV/PhiC31 [26]), retroviruses (E1-deleted AdV/RV [27,28]), or adeno-associated virus (HDAdV/AAV [29–31]) (Table 2.). A summary of all integrating hybrid vectors based on adenovirus as a delivery vehicle is displayed in Figure 2. The following section will provide further details on each single hybrid vector system.

### 2.1. Adenovirus/AAV hybrid vectors

One of the earliest studies unified the advantageous gene delivery features of a first generation E1-deleted adenoviral vector with the AAV genome simply for production of recombinant AAV (rAAV) [32]. Afterwards Ad/AAV hybrids were mainly generated for developing novel gene therapy vectors and in particular also for achieving site-specific integration into the host genome. Responsible for the unique ability of AAV to integrate into a specific locus on human chromosome 19q13.3-qtr (called AAVS1) are the inverted terminal repeat (ITR) *in cis* and the Rep proteins (Rep68 or Rep78) *in trans* [33–35]. Rep68/78 bind to the Rep-binding site located on both, the AAV genome and the genomic AAVS1 pre-integration site. Subsequently, a non-homologous deletion-insertion recombination results in the integration of 2–4 copies of the AAV-ITR-cassette [33,35]. In the absence of Rep proteins, AAV genomes show a bias towards integration into actively transcribed genes [36]. Notably, rAAV vector genomes do not rely on integration into the host genome. In fact, there is evidence that integrated as well as extrachromosomal rAAV DNA molecules are responsible for transgene expression [37].

The molecular design of Ad/AAV hybrid vectors is very diverse. Basically, they can be divided into two groups of which one group utilizes AAV-Rep protein for site-specific integration and the other group is based on Rep-independent rAAV genome integration (Figure 2. i). With respect to the latter group, rearranged Ad/AAV vector genomes (delta Ad/AAV genomes) [38,39], containing solely the transgene flanked by AAV-ITRs in an HDAdV genome showed integration at random sites in the host genome [38,40]. Other strategies were based on packaging of rAAV genomes into adenoviral capsids (AAV/adenovirus hybrid vectors) by including the adenoviral packaging signal in the AAV genome [41,42].

The first Ad/AAV hybrid vector for Rep-mediated site-specific integration at AAVS1 on human chromosome 19 was developed by Recchia *et al.* [31]. This system was based on a two-vector HDAdV system. One vector carried the Rep78 gene under control of either the T7 or alpha-1-antitrypsin liver-specific promoters and the other vector contained an AAV-ITR-flanked transgene. Up to 35% of integration events into AAVS1 in HepG2 cells were detected after co-infection with these two HDAdVs. However, Rep protein significantly affected the production of the adenovirus encoding the elements of the AAV integration machinery [31]. Therefore, further improvements like the Cre/loxP-expression-switching system to regulate the expression of the *rep* gene were generated [134]. Later on, Recchia and coworkers improved the Ad/AAV hybrid vector by using a tetracycline-regulated *rep* expression system. Notably, in this study the authors could also show site-specific integration into the AAVS1 site in AAVS1 transgenic mice and in human primary cells [43].

To broaden the tropism of adenovirus/AAV hybrid vectors, Gonçalves *et al.* described the generation of dual Ad/AAV hybrid vectors with fiber-modified adenoviral capsids and with all the AAV *cis*- and *trans*-acting elements needed for locus-specific insertion of exogenous DNA [30]. To avoid the well-described interference of the large AAV Rep proteins (*i.e.*, Rep78 and Rep68) on adenovirus DNA replication, they devised a *flp* recombinase-dependent gene switch module to repress and activate *rep68* expression in producer and target cells, respectively [30]. Studies in HeLa cells showed an increased stable transduction level of the transgene and AAVS1-targeted integration of vector DNA.

### 2.2. Adenovirus/Sleeping Beauty transposase hybrid vectors

This hybrid vector couples high-efficient gene delivery of HDAdVs with the Sleeping Beauty (SB) transposase integration machinery [25]. SB is a synthetic transposable element reconstructed from the fish genome and belongs to the Tc1/mariner family of transposable elements. It mediates insertion by a ‘cut-and-paste’ mechanism into TA sites of the host genome [1]. The transposition process requires the binding of the SB-transposase to short direct-repeats embedded in the terminal repeats (IR) flanking the transposable elements [1] (Figure 2. iii). Generally, for gene transfer purposes a two-component system is used for mobilization of the transposon from the episomal transposon-donor vector. A second vector provides the transposable element *in trans*. Recently, different hyperactive variants of SB transposase (SB11 [144], HSB5 [124], SB100X [48]) were developed by mutational analysis screens. Geurts *et al.* [144] developed the first improved SB transposase (SB11) which enhanced transposition efficiencies about threefold [144]. Further improvements of this genetic element for somatic integration from Yant and colleagues resulted in the development of the HSB5 transposase with 10-fold increased activities compared to the wild type SB transposase [124]. At present the most hyperactive transposase (SB100X) displays 100-fold enhanced integration activity [48].

SB has been shown to provide long-term transgene expression *in vitro* with a random integration profile [44]. In detail, a recent study has shown, in contrast to most retroviral-based vectors that SB integrates fairly randomly in mammalian cells with a low preference for actively transcribed genes [45]. Besides *in vitro* studies for transposition, plasmid-based transposons carrying therapeutic transgenes were delivered into mice [46,47], transgenic mice were produced and primary hematopoietic stem cells were genetically modified [48].

Since efficient *in vivo* delivery of the SB transposase system utilizing naked DNA remains a challenging task, much effort was spent on designing virus-based delivery technologies. One of the first delivery methods was predicated on recombinant adenovirus. Yant and coworkers developed a two-component HDAdV-SB transposon system [25]. For the rational design of this hybrid vectors system, it has to be mentioned that one precondition for efficient SB-mediated integration is to supply a circular substrate [25]. Since adenoviral vector genomes predominantly persist as linear, episomal vector genomes, the SB substrate needs to be circularized and therefore mobilized from the adenoviral vector genome [7]. In this case, the transposon was achieved by Flp recombination. One vector carried a human coagulation factor IX (hFIX) transposon flanked by Flp recombinase recognition target (FRT) sites for circularization from the adenoviral vector. The second vector expressed Flp and SB transposase. Administration of these vectors resulted in the generation of transposon circles and random SB-mediated transposition of the hFIX transgene into the host genome, mediating hFIX expression for more than 6 months despite extensive liver regeneration after multiple partial hepatectomies [25].

### 2.3. Adenovirus/PhiC31 hybrid vectors

The integrase from the Streptomyces phage PhiC31 was described in 1991 for the first time [49]. In contrast to SB, PhiC31 was shown to mediate an unidirectional site-specific recombination between two DNA recognition sequences, the phage attachment site (*attP*) and the bacterial attachment site (*attB*). Expressed under the control of a mammalian promoter, PhiC31 integrase mediates integration of extrachromosomal plasmids bearing an *attB* site into a limited number of *‘pseudo-attP’* sites present in native mammalian genomes [2]. A remarkable feature of the PhiC31 integrase for gene therapy is the fact that integration is limited to “hot spot” sites with up to 15% specificity in the mammalian genome [50–53]. This feature of PhiC31 integrase potentially decreases the risk of insertional mutagenesis.

With respect to integrating viral hybrid vectors, our group utilized HDAdV as an efficient delivery vehicle in concert with the integration machinery of integrase PhiC31 for stable transduction and limited integration sites [26]. The strategy and the design of this system are schematically displayed in Figure 2. iv. Similar to the Ad/SB hybrid, a two-vector system was designed, in which the transgene expression cassette is circularized from the HDAdV genome by Flp-mediated recombination, followed by PhiC31-mediated somatic integration. This vector system was evaluated in mice and integration of the transgene expression cassette from the adenoviral vector resulted in 5-fold higher transgene expression levels and integration into the previously described hot spot mpsL1 present in murine liver [26,52]. It is of note that recent results may indicate, that Flp-mediated circularization from the linear adenoviral vector genome may not even be required for PhiC31 integrase-mediated integration. In contrast to the SB transposase integration machinery, linear DNA substrates can serve as substrates for PhiC31-mediated integration [26].

### 2.4. Adenovirus/retrotransposon hybrid vectors

Another strategy for somatic integration of the transgene from an adenoviral vector is the use of retrotransposition based on the human long interspersed element-1 (L1). L1 sequences are retrotransposons without LTR, which comprise approximately 17% of the human genome. The integration mechanism is initiated by L1-RNA expression and its transport into the cytoplasm where the ribonucleoprotein particle (RNP) is formed. Subsequently this RNP is transported into the nucleus via an unknown mechanism followed by L1-endonuclease mediated cleavage of a consensus target DNA in the host genome and subsequent integration.

The first chimeric system combining HDAdV and L1 retrotransposons has been elaborated by Soifer *et al.* [54]. Efficient adenovirus-mediated delivery of the L1 element into cultured human cells resulted in retrotransposition and stable integration of the transgene [24,54] (Figure 2. v). Kubo and colleagues developed a second-generation adenovirus-L1 retrotransposon hybrid virus and demonstrated, that this L1 element could efficiently retrotranspose in non-dividing cells arrested in the G1/S phase of the cell cycle and in differentiated primary human somatic cells [55].

### 2.5. Adenovirus/retrovirus hybrid vectors

Recently, several research groups designed hybrid vectors that included both retroviral and adenoviral elements [27,28,56–58,141–143]. The first reports by Feng *et al.* [58] and Caplen *et al.* [56] demonstrated the feasibility of this strategy by showing proviral integration in a nude mouse tumor model system. The original idea of these approaches was to generate chimeric adenoviral vectors to function as transient retroviral producer cells capable of infecting neighbouring cells. Similar to the latter approaches, Soifer and colleagues used HDAdV as a carrier to deliver a fully functional retrovirus vector [143]. They demonstrated that this HDAdV/RV hybrid vector mediates highly efficient gene delivery and permanent integration of transgenes through a two-stage mechanism. With respect to lentiviral vectors, Kubo and colleagues developed a hybrid vector, in which a HDAdV was used as a carrier of the LV production machinery [142]. However, in contrast to the HDAdV/RV hybrid vector, the latter vector transduces both dividing and nondividing cells in a TET-inducible manner. Furthermore, Kuate *et al.* [141] took advantage of the high transduction efficiencies of adenoviral vectors for generation of a novel packaging system for lentiviral vectors. This system is based on transient expression of packaging genes by recombinant adenoviruses.

The first Ad/RV hybrid vector for somatic integration of a transgene expressing cassette flanked by the retroviral long-terminal repeats (LTRs) was generated by Zheng and co-workers [28]. They constructed an adenoviral vector (AdLTR–*luc*) carrying the 5′ and 3′ LTR sequences from murine leukemia virus (MLV) and a luciferase reporter gene. Gene expression was observed in cultured cells *in vitro* and in submandibular gland, cortex, and caudate nucleus for as long as three months *in vivo*. The vector integrated randomly into the genome of both dividing and non-dividing cells as determined by fluorescence *in situ* hybridization (10–15% of cells *in vitro* and 5% in rat spleen *in vivo*).

Two years later another Ad/RV hybrid vector was introduced by Murphy *et al.* [27]. In contrast to the earlier study, somatic integration was mediated by retroviral integrase. The strategy was based on excision by the Cre recombinase of a provirus circle from the adenoviral vector containing the retroviral LTR. In the presence of retroviral Gag and Pol proteins, the excised circle was integrated into the host genome by an integrase-directed mechanism. This resulted in a random integration process. Both strategies of Ad/RV hybrid vectors (with and without integrase-mediated integration) are schematically shown in Figure 2. ii.

In 2004 an adenovirus/foamy virus (Ad/FV) hybrid vector was developed [59]. For foamy virus (FV), a member of the retrovirus family, it is believed that there are no known pathogenicities associated with infection. Therefore its integration machinery is attractive for stable gene transfer. The Ad/FV hybrid vector, which resulted into 70% of stably transduced cells, contained a complete expression cassette for FV gene products (Gag, Pol, and Env) necessary to generate an infectious extracellular particle.

## 3. HSV-1 based hybrid vectors for somatic integration

Herpes simplex virus (HSV) belongs to the subfamily of alpha herpesvirus viridae within the family of herpesviridae. It is a member of the large group of enveloped viruses and contains a genome of approximately 152 kb in size [6]. HSV vectors for gene therapeutic approaches are usually based on the HSV serotype 1 (HSV-1). In the clinic HSV-1 typically causes the cold sore, but rarely also acute hepatitis, kerato-conjunctivitis or meningo-encephalitis [6]. HSV-1 displays a strong natural tropism for neurons and therefore, these vectors were mainly used in gene therapeutic approaches involving the nervous system.

Two fundamentally different HSV-1-based vector systems were developed, recombinant HSV-1 vectors and HSV-1 amplicons. Whereas recombinant replication-competent HSV-1 vectors are mainly used as oncolytic viruses, amplicon vectors with major genome deletions are mainly used as gene transfer vectors [60].

HSV-1 amplicon vectors are helper-dependent vectors which carry a concatemeric form of a DNA plasmid containing for the HSV-1 origin of replication (*ori*), the HSV-1 packaging signal (*pac*) and the gene of interest. These sequences enable packaging in infectious HSV-1 particle [61]. Herpes virus amplicon vectors were used as delivery vectors for gene therapy mainly because of their high capacity, their low cytotoxicity and the ability of the virions to infected a wide range of dividing and non-diving cells, especially neuronal cells [62]. Notably, in contrast to recombinant adenoviral vector genomes, the HSV genome circularizes and is maintained within the nucleus as a double-stranded DNA genome. Recently, it was shown that these HSV-1 amplicon vectors can be used to deliver DNA constructs that are larger than 100 kb [63]. However, the major problem of amplicon vectors is the helper virus free production. At present a method combining the total absence of contaminating helper particles with the ability to produce very large amounts of amplicon particles is not available [64].

**Figure 3.**
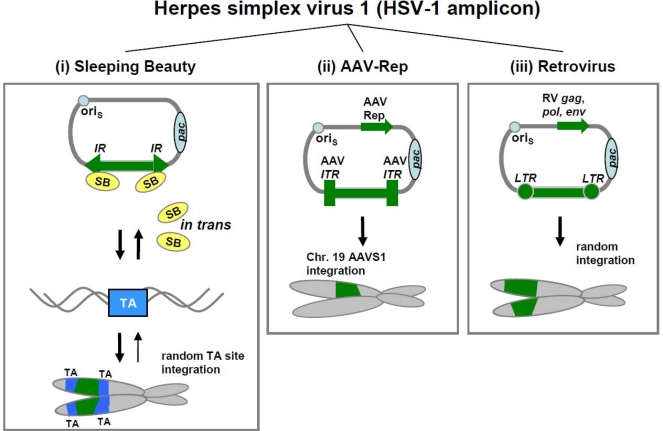
Mechanisms of herpes simplex virus 1 (HSV-1) amplicon based integrating hybrid vector systems. HSV-1 amplicon systems carry the HSV-1 origin of replication (*ori_s_*), the HSV-1 packaging signal (*pac*), and the transgene flanked by specific elements necessary for the indicated integration machinery. The DNA is packaged as concatemers into the HSV-1 virions. (i) For Sleeping Beauty (SB) transposase mediated integration the transgene is flanked by inverted repeats (IRs). The SB protein, expressed *in trans*, binds to the IRs excises the transposon and results in reversible random integration in TA-sites in the host genome. (ii) The HSV-1/AAV-Rep amplicon hybrid vector leads to site-specific insertion of transgenes flanked by AAV inverted terminal repeats (ITR) into the AAVS1 site of human chromosome 19. The AAV-Rep protein is produced *in cis*. (iii) In case of HSV-1/retrovirus (RV) amplicon vectors the transgene incorporated between two long terminal repeats (LTRs) is translocated via the retroviral proteins Gag, Pol and Env. This leads to a random integration pattern.

Nevertheless, amplicon vectors are used in a large set of experimental applications. However, the episomal nature of the HSV-1 amplicon limited the duration of the transgene expression and therefore the development of amplicon with the ability to integrate the transgene into the host genome was of great interest to research community. The following section will provide an overview of the molecular setup of HSV-1 amplicon/AAV hybrid vectors, HSV-1 amplicon/SB transposon hybrid vectors, and HSV-1 amplicon/retrovirus hybrid vectors. The basic molecular setup of these hybrid vectors is depicted in Figure 3.

### 3.1. HSV-1 amplicon/AAV hybrid vectors

The first studies incorporated AAV ITRs and Rep functions into HSV-1 amplicon vectors to induce integration of the transgene expression cassette into specific sites of host chromosomes as shown in Figure 3. ii [65,66]. In initial experiments from Johnston and colleagues an amplicon vector was constructed containing the *lacZ* reporter gene under control of the cytomegalovirus IE1 promoter (CMV IE1) flanked by AAV ITR sequences, to facilitate genomic integration of this cassette in the host genome [67]. Constructs were generated with and without AAV Rep coding sequences. In the presence of Rep proteins transgene retention was supported and after transduction of human glioma cells expression for more than two weeks could be detected [67]. Hybrid vector mediated site-specific integration *in vivo* was also provided by Breakefield and colleagues. These authors utilized the HSV-1/AAV hybrid amplicon vector to successfully target the integration of an ataxia-telangiectasia mutated (ATM)-encoding cDNA into the AAVS1 locus of AAVS1-bearing transgenic mice [68].

Other studies have also shown that hybrid amplicon vectors with transgenes flanked by ITR sequences from AAV packaged in HSV-1 virions can mediate integration of transgenes into the AAVS1 site in human cells when Rep was provided [65,66,69,70]. However, in these studies expression of Rep protein had toxic effects with respect to HSV-1 replication and a low packaging efficiency during HSV-1/AAV amplicon production resulting in low titers was observed [65,66].

To improve the production, Lui *et al.* added loxP sites into the HSV-1/AAV hybrid amplicon, with the intent to excise the ITR-transgene-containing fragment from the amplicon concatemer by Cre-loxP-mediated recombination. As a result they yielded titers comparable to those of standard HSV-1 amplicons [69]. For small transgene cassettes (4–5 kb) these vectors can achieve genomic integration rates of 10–30% of infected human cells in culture with about 50% of these integration events occurring specifically at the AAVS1 locus [65,66]. Also large functional inserts of genes (100 kb) were successfully integrated into the AAVS1 site using an HSV-1/AAV hybrid vector, but efficiency was reduced in comparison to smaller transgene sequences [70].

### 3.2. HSV-1 amplicon/SB transposase hybrid vectors

A second strategy to induce integration into host chromosomes of transgenes delivered by amplicon vectors is the use of DNA transposons like SB transposon (Figure 3. i). This platform consists of two vectors, one carrying a viral/cellular promoter driven transgene flanked by SB inverted repeats and the second vector harboring the SB transposase gene under the control of the HSV immediate-early 4/5 gene promoter. Co-delivery of these vectors facilitated long-term expression by random integration *in vivo* and *in utero* and transmission in dividing cells after integration of the IR flanked transgene into the mouse genome [71]. In baby hamster kidney (BKH) cells, the authors demonstrated an approximately 25-fold increased transduction efficiency using the integration-competent amplicon system compared to conventional amplicons.

### 3.3. HSV-1 amplicon/retrovirus (RV) hybrid vectors

HSV-1/RV hybrid vectors can also provide prolonged transgene expression by integration of the transgene into the host genome (Figure 3. iii). Initially, this HSV-1/RV amplicon vector based on murine leukemia virus (MLV) was constructed for generation of infectious retroviral particles [72]. In addition to the packaging gene and replication origin of HSV-1, this amplicon vector contained MLV LTRs flanking the transgene expression cassette and in a separated cassette *gag*, *pol* and *env* proteins were encoded [73]. De Felipe *et al*. reported the construction of a HSV-1/RV hybrid vector that exhibits up to 50-fold higher transgene integration efficiency compared to vectors containing solely HSV-1 components [74]. Efficient integration of a retroviral transgene cassette encoding the Pac protein in human cells required expression of the MLV *gag-pol* genes, but in murine cells, also endogenous activities could mediate integration, albeit at a lower level. Gene delivery was equally efficient in BHK, a cell line resistant to retroviral infection, and in addition transgene retention and expression were stable for at least one month in Hs683 human glioma cells.

## 4. Retrovirus-based hybrid vectors for somatic integration

**Figure 4.**
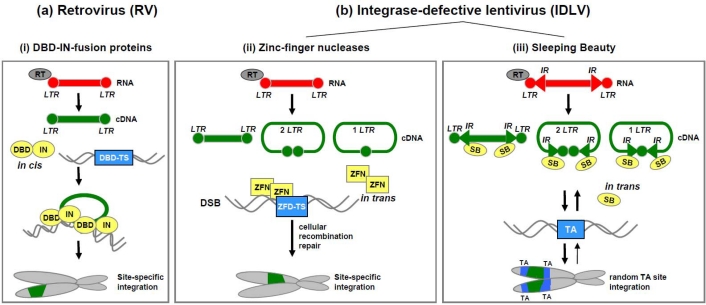
Strategies of retroviral integrating hybrid vector systems. Retroviral vector systems are (a) based on integrase-expressing retroviruses (RVs) or (b) on integrase-defective lentiviruses (IDLVs) as transgene delivery machineries. Retroviral RNA, flanked by long terminal repeats (LTRs) is reverse transcribed to cDNA by the reverse transcriptase (RT) in the host cells. The transgene insertion occurs like indicated in the scheme (i–iii). (i) Retroviral vectors express integrase (IN) proteins *in cis*, which are fused to specific DNA-binding domains (DBD) and trigger insertion in specific DBD target sites (TS) in the genome. (ii) For site-specific targeting of the transgene, which is delivered by IDLVs, zinc-finger nucleases (ZFN) are utilized. The zinc-finger-domain (ZFD) of the ZFN binds to the zinc-finger domain target site (ZFD-TS) and mediates double strand breaks (DSB) at this specific site in the genome. Subsequently, the transgene is integrated and the DNA repaired. (iii) Hybrid vectors based on IDLVs harbour recognition sites (inverted repeats, IRs) for Sleeping Beauty (SB) transposase binding. The viral cDNA can form a linear structure with 2-LTR motifs or circular formations with 2 LTRs or 1 LTR. SB expressed *in trans* mediates the insertion of the transgene into TA sites of the host genome.

Integrating retroviral vectors have been used for delivery of transgenes for over 20 years of gene therapy (Figure 4). Retroviral vectors based on MLV were the first types of vectors used in gene transfer and in clinical trials. Today, the list of retroviral vector types for experimental and therapeutic applications has been broadly extended. Besides MLV also vectors based on human immunodeficiency virus (HIV), avian sarcoma-leukosis virus (ASLV), and foamy virus (FV) are utilized for transduction of therapeutic DNA into target cells. Notably, preclinical and clinical gene transfer studies were pioneered by MLV-based retroviral vectors in the early nineties. In fact, the first clinical trial in gene therapy was performed with retroviral vectors in two girls affected by a form of severe combined immune deficiency characterized by adenosine deaminase deficiency [75].

The most desirable advantage of this vector system is the ability of irreversible integration into the host genome. For medical application this system introduces a foreign DNA efficiently and easily into specific target cells. The molecular organisation of the retroviral genome consists of three open reading frames (ORFs) *gag, pol* and *env* encoding matrix-, capsid-, envelope proteins, as well as viral enzymes like protease, reverse transriptase and the viral integrase. These genes are flanked by long terminal repeats (LTRs), responsible i.a. for the regulation of gene expression. The *psi* element near the 5′end of the viral genome encodes the packaging signal for the virus. For complex retroviruses additional accessory proteins are synthesized after distinct complex splicing mechanism, which alter within the retroviral genus and are essential for viral replication *in vivo*. For instance HIV-1 generates spliced RNA encoding Tat and Rev as well as Vpr, Vif and Nef [76].

The mechanism of integration involving participating viral and host proteins is well understood. Regarding biosafety in gene therapy, the integration site pattern is a topic of interest. Retroviral integrase, which is encoded by the virus, is essential for the transgene insertion process. In concert with viral DNA and genomic host DNA, this enzyme builds a preintegration complex (PIC) with the LTRs *in vivo.* The integration process occurs via processing genomic DNA, joining DNA ends and postintegration repair of strand breaks [77–81].

The site selection of integration restricts no specific target sequences in the genome, but it does not occur randomly. Within the retroviral family the integration patterns differ significantly [82]. For HIV, translocation preferentially occurs into transcriptionally active genes [83]. MLV infections show a strong bias towards transcription start sites and CpG islands [84,85]. Also FV and ASLV show a distinct integration pattern. Regarding FV Trobridge and colleagues revealed that FV did not integrate preferentially within genes but with a considerable preference for CpG islands and a low preference for integration near transcription start sites [86]. For ASLV it was found that it does neither favour integration near transcription start sites, nor does it strongly favour active genes [82,87].

Because of integration patterns it has been shown that insertion of retroviral DNA can be associated with the induction of leukemia in animal models as well as in human therapy trials utilizing MLV based vectors for stable gene transfer [88,89]. Therefore, it would be desirable for the usage of retrovirus in a gene therapeutic manner, if integrase activity is linked to predetermined and safe sequences in the host genome [90]. Herein, we summarize the most important technologies for changing the integration profile of retroviral vectors by developing RV-based hybrid vectors. The design of these hybrid vectors is displayed schematically in Figure 4.

### 4.1. Retrovirus/integrase-fusion protein hybrid vectors

Several approaches deal with the construction of integrase (IN) -fusion proteins based on specific targeting strategies in the host genome (Figure 4. i). DNA-binding domains (DBD) derived from *E. coli* LexA repressor protein, from the phage λ repressor or a murine transcription factor Zif268 were fused either N- or C- terminally to the viral integrase of HIV or ASLV [91–94]. These fusion proteins enable site-specific integration nearby their DNA target sites *in vitro*. More specific targeting into the host genome occurs with fusing the integrase to a designed polydactyl zinc-finger motif (E2C) enabling the recognition of a specific 18-bp long attachment sequence [95]. These synthetic proteins are derived from Cys2-His2 zinc-finger domains and provide site-specific integration *in vitro* [4]. For this E2C/IN-fusion protein it is known that the recognition sequence is located within the 5′untranslated region of the *erb2*-gene of the chromosome 17q12 [96]. Incorporation of the IN-E2C fusion protein in an infectious virion was performed by providing the protein *in trans*. Evaluation of integration pattern provided a 10-fold higher efficiency regarding the *erb2*-region compared to the wild type virion [4,95,97].

### 4.2. Lentivirus/zinc-finger nuclease hybrid vectors

Another strategy for targeting retroviral vectors to specific genome sites is to combine engineered zinc-finger nucleases (ZFNs) for site-specific integration and non-integrating lentiviral vectors (IDLVs) for delivery (Figure 4. ii). Development of IDLVs is due to the inactivation of the viral integrase by mutating the catalytic domain for integration without effecting other protein functions like reverse transcriptase activity [98]. During infection IDLVs accumulate episomal intermediates such as LTR-circles within a cell and it was shown that these LTR circles mediate short-term transgene expression within the transduced cell [99,100].

Lombardo and colleagues utilized IDLVs to deliver ZFNs for site-specific integration and to incorporate template DNA for gene correction into target cells [101]. These ZFN induce double strand breaks at the desired DNA sequences, allowing the DNA template to integrate at this particular site [102,103]. After binding of the transgene encoding sequence, the DNA break is subsequently repaired by the cellular repair mechanism, which involves either non-homologous end joining or homologous recombination [104].

After co-transduction of different cell types with IDLVs containing all elements for ZFN-mediated site-specific integration, Lombardo *et al*. observed that up to 50% of the human stem cells were stably transduced [101]. Importantly, these cells showed gene addition at the target sites of the ZFN. This method specifies the integration sites by the zinc-finger motifs.

### 4.3. Lentivirus/Sleeping Beauty transposase hybrid vectors

Very recently, there were two reports utilizing SB transposase in concert with IDLVs [105,106]. Both studies took advantage of the IDLV technology for high transduction efficiencies and the SB transposase technology for efficient somatic integration of the transgene. After infection with these IDLVs, transposition occurs from episomal viral DNA substrates with SB transposase supplied *in trans* (Figure 4. iii). However, the study by Staunstrup and colleagues [105] was based on the newest and most hyperactive SB transposase version SB100X and the study by Vink *et al.* [106] was based on the SB transposase mutant SB11 displaying significantly lower integration efficiencies.

Notably, co-transfected DNA as well as co-transduced IDLVs can serve as the source of active SB transposase. In contrast to conventionally used lentiviral vectors, this system resulted into a random genomic integration profile and it avoids insertion into active gene regions. These studies showed that replacement of the viral integration machinery with non-viral mediators of integration represents a new and promising platform of lentiviral vectors with an altered integration profile.

## 5. Safety issues

There are two main starting points for improving the safety of viral hybrid vectors. An optimal hybrid vector should account for site-specific integration into a safe locus within the mammalian genome without off-target effects and it should result in no or limited immunological responses and induce toxic side effects. Therefore, although vectors described in this article have major potential, there are also limitations for each single vector system which need to be addressed. The following paragraphs will discuss the safety profile of the various vectors presented in this article.

### 5.1. Safety and genotoxicity of integration machineries used within hybrid vectors

With respect to viral hybrid vectors utilizing SB transposase for somatic integration, one concern may be the random integration pattern with approximately 30% integration into actively transcribed chromosomal regions [45]. Although SB transposase represents a safer option compared to retroviral vectors which predominantly integrate into actively transcribed genes (up to 75% of all integration events occur in transcriptionally active genes), it can not be ruled out at present that even a random integration pattern may result in genotoxicity. Nevertheless, the SB transposase system holds great promises for gene therapy and it did not come as a surprise that in 2008 the first clinical trial was initiated. Herein, patients with CD19^+^ B-lymphoid malignancies will be treated with genetically altered T cells [107] and the SB transposase system will be used to express a CD19-specific chimeric antigene receptor in autologous *ex vivo* expanded T cells.

Considering the fact that PhiC31 integrase can result in up to 15% specificity of somatic integration [50,51], this system also represents an attractive tool for genome engineering and therapeutic applications. However, there are important safety issues which need to be addressed before pursuing this system in preclinical or even clinical studies. One limiting factor is that PhiC31-mediated integration preferentially occurs into intron regions of actively transcribed regions [50,51]. Another concern is that deletions of DNA at the junctions of chromosomal DNA and the attB attachment sites occur. Most importantly, in-depth molecular analyses revealed that chromosomal rearrangements are induced as a consequence of PhiC31 activity within treated cells [50,51,69,108]. Our previous study revealed that of all analysed integration sites, 15% were found to contain a transgene which was flanked by DNA sequences originated from two different chromosomes [51]. Notably, until now chromosomal aberrations and deletions were not found after SB- and retrovirus-mediated integration of transgenes. Only for recombinant AAV lacking all viral coding sequences (*rep* and *cap*) frequent chromosomal deletions were detected [36].

ZFNs for site-specific genomic engineering and gene correction represent one of the most advanced technologies. In particular, site-specific integration into a potentially safe harbour of the host genome which is not associated with genotoxicity represents a potential solution. However, there is still a risk for off-target effects as a consequence of unspecific nuclease activity within transduced cells and there is evidence that this in turn may lead to cytotoxicity and apoptosis [109,110]. To address these issues, structure-based approaches for improving specificity of ZFNs were performed [111,112]. By preventing homodimerization of ZFNs, specificity could be increased and genotoxicity was reduced. Nevertheless, as long as off-target effects can not be completely circumvented, this system will require improvements. Besides optimizing the structural properties of ZFNs, the actual amount of zinc-finger nuclease molecules produced within a cell and the duration of nuclease expression may be an important factor influencing safety issues of this technology.

Besides ZFNs and PhiC31 for site-specific integration, AAV-Rep-protein mediated site-specific integration into the human locus AAVS1 on human chromosome 19 (chromosome 19q13.4) seems an appealing approach. Having a closer look at the AAVS1 locus and its consequences after AAV provirus integration, it was found that amplification and rearrangements at the AAVS1 locus occurred due to site-specific provirus integration [113]. Furthermore a number of AAV integration hotspots were identified in the first exon/intron regions of a genomic sequence encoding the p85 protein sharing similarity with the myosin binding subunit MBS130 [114]. At present it is not known whether these changes on a genomic level can result into any genotoxicity. Previous studies indicated that there is an appreciable prevalence of neutralizing antibodies to AAV in the human population (up to 30% for AAV2) [115,116], hinting towards noteworthy provirus integration at the AAVS1 site. Thus, one may speculate that the probability of unwanted side effects is low, because there is no known pathogenicity associated with AAV infection.

However, there is evidence that Rep-proteins responsible for integration at AAVS1 have negative effects on viral replication of HIV, adenovirus and HSV-1[31,65,66,117]. Furthermore, there are hints that Rep-proteins, if expressed at high levels, are associated with cytotoxic and/or cytostatic effects [118,119]. These are concerns with need to be taken into account when working with this system.

It remains to be emphasized that with all three systems for targeted integration (ZFNs, phiC31, and AAV/Rep) also variable levels of random integration can be observed. Efficiencies of targeted integration very from 7% to 90% with various levels of random integration and thus, at present genotoxic effects can not be excluded for any of these integration machineries.

Furthermore, it is important to mention that in contrast to naturally integrating vectors such as retroviruses, integration frequencies of integrating hybrid vectors may be more difficult to control, because these are determined by several factors including cellular entry of two vectors into one cell and subsequent chromosomal integration. These features make hybrid vector systems more complex and therefore also more unpredictable, which in turn may increase the risk of insertional mutagenesis.

### 5.2. Assays to evaluate genotoxicity

Last but not least it may be helpful to develop standardized procedures to control reliability and safety of each single integrating vector approach. Testing the likelihood of insertional mutagenesis may be addressed by performing large-scale sequencing analysis of insertion sites after stable gene transfer. This would give us an idea of how many times the therapeutic DNA integrates into chromosomal regions with a high risk for genotoxicity. Moreover, it is possible to evaluate the oncogenic potential after viral transduction with a recently developed *in vitro* assay [120] or in a tumor-prone mouse model [121].

### 5.3. Safety of gene transfer vehicles utilized in the context of hybrid vectors

When using viral vectors for gene transfer, it is important to point out that not only genotoxicity but also immunological side effects associated with the incoming viral particle itself, the viral proteins utilized for somatic integration or with new transgene products that might be recognized as foreign, may be a considerable safety concern. Adenovirus vectors are the most immunogenic of all the viral vector groups but since the discussion of these unwanted side effects for all viral delivery vehicles described in this review would go beyond the scope of this article, please refer to other articles specifically dealing with these issues for more detail [135–137].

Unwanted transduction of non-target cells is another issue of concern. Except for HSV-1, a virus which displays a strong neurotropism, retroviral and adenoviral vectors are characterized by a broad tropism. Thus, administration of these vectors *in vivo* may not only result in stable transfection of target cells, but also in an “overspill” towards infection of other, unwanted cells.

It is of note, that the viral- (AAV/Rep, retrovirus integrase) as well the non-viral (SB, Flp, ZFN, phiC31) proteins used for integration in the context of hybrid vectors may also cause cellular immune responses. However, more in depth studies have to be performed and assays need to be developed to address these issues in more detail.

## 6. Outlook

Recapitulating the list of existing viral hybrid vectors described in this review, there are vector-recombinase-combinations which have not been explored (see also Table 1.). Completing the possible combinations by mixing and matching the viral delivery vehicles and the currently established tools for somatic integration may be achieved within the next years. But many hurdles remain to overcome. For instance rAAV vectors and rAdV for delivery of ZFNs and the respective donor vector providing the integration machineries may be an interesting approach to pursue. Also, delivery of SB transposase or PhiC31 integrase utilizing AAV vectors may be another alternative to follow. Furthermore, HSV-1 based therapeutic DNA delivery into neuronal cells may be explored for PhiC31 integrase, but also delivery of the ZFN-technology represent an interesting option to look into more closely.

### 6.1. Towards a “one-vector system”

One important challenge which certainly needs to be addressed in the near future is the complexity of currently available hybrid vector systems. The majority of them are based on “two-vector strategies”. Disadvantages of these approaches are obvious, because they rely on simultaneous co-transduction of one cell with two vectors. This experimental setting can be successful for *in vitro* approaches because the viral dose can be increased more easily and the ratio of both vectors can be mixed and matched, but for cells with naturally low transduction rates for the respective virus and for *in vivo* approaches this may be problematic with respect to negative side effects.

We believe that exploring a “one-vector strategy” may be one potential solution. In more detail, this means that all components for somatic integration (therapeutic DNA and the respective recombinase) are combined in one vector genome. However, as already shown for Ad/AAV hybrid vectors, it may be troublesome if the recombinase and the correspondent donor-DNA co-exist in the identical vector genome. Expression of the recombinase during vector production may significantly decrease viral titers or it may even destroy the vector due to unwanted removing and/or integration of the transgene DNA into the host genome of the vector-producer cell line. Thinking about hybrid-vectors utilizing AAV as a delivery vehicle, the relatively low packaging capacity of recombinant AAV (4.7 kb) may be problematic for insertion of all genetic elements into a single vector. Interestingly, it was suggested very recently that AAV serotype 5 (AAV5) capsids may be able to carry a genetic cargo of up to 8.7 kb [122]. This feature of the AAV5 capsid may be an option for a “single-AAV-vector” approach.

### 6.2. Heading for targeted integration

One essential improvement required before exploring these hybrid vectors in a clinical setting will be the development of recombinases for effective site-specific integration into a safe harbour without any side effects. First steps towards this goal have been initiated. For instance ZFNs specifically targeting the AAVS1 locus as a potential safe harbour have been generated in collaboration with Sangamo BioSciences [123]. In any case, one important question which remains to be answered is whether the AAVS1 locus is truly a safe harbour for therapeutic DNA insertion.

Besides ZFNs for targeted insertion, there have been attempts to generate SB transposase fusion proteins for site-specific integration. In that case, specific DNA-binding domains were linked to SB transposase redirecting the transposase towards one integration site [124,125]. Although efficiencies of these approaches were low, with further improvements this idea may remain a valuable option in view of recently generated hyperactive SB variants [48].

Regarding PhiC31 integrase, there was only one recent report on improving integration efficiencies and specificities by performing a mutagenesis screen of the catalytic domain of the PhiC31 protein [126]. However, with respect to specificity it may be also interesting to conduct a mutagenesis screen of the proposed PhiC31 integrase DNA-binding domain. Furthermore, similar to other approaches described above, it may be interesting to develop a PhiC31-zinc-finger fusion protein for redirecting target site selection of the PhiC31 protein.

### 6.3. Surface engineering and retargeting of viral hybrid vectors

To essentially broaden applications of adenovirus-, retrovirus-, HSV-1, and AAV-based hybrid vectors, it will be of interest to combine these vector types with novel targeting strategies which were developed for basically all viral delivery vehicles discussed in this review. For more details about targeting strategies for adenoviral, AAV, HSV and retroviral vectors please refer to other review articles specifically discussing viral vector targeting and virus tropisms (e.g., [127–132]).

Last but not least, it is important to explore these hybrid vector systems in larger animals, because the toxicity profile may be species dependent. History has taught us that the performance of a viral vector strongly differs between mice, larger animals and humans. At present, it remains an unanswered question whether combining the best of two worlds in viral hybrid vectors will provide a valuable option for therapeutic and experimental gene transfer approaches. Looking into the future, we believe that customized viral hybrid vectors which address the single needs of a certain genetic or acquired disease will play an important role.

## 7. Conclusion

For successful treatment of various genetic diseases genomic insertion of therapeutic DNA is required for achieving long-term therapeutic effects. In this review we provided a state-of-the-art overview of integrating hybrid vectors. We believe that these vector types have a high potential for a variety of clinical applications for which conventional vectors may not be suited, because hybrid vectors may be specifically designed for a certain disease and its individual needs. However, at the time the appropriate answer to the original question raised in the title of this review article (Viral hybrid vectors for somatic integration - are they really the better solution?) should be that for certain diseases and applications the current versions of integrating viral hybrid may be the better solution but for others they are not.

For instance, due to the complexity of currently available hybrid vector systems, they may not be suited for *in vivo* applications in their current forms. We believe that gene therapy of monogenic diseases like hemophilia B would most likely not benefit from integrating viral hybrid vectors, because non-integrating HDAdV and AAV vectors were already shown to result in long-term expression of the human coagulation factor IX in mice and dogs [23,146]. Furthermore, it remains to be seen whether integrating hybrid vectors that are based on SB transposases, ZFNs or other integrases will indeed show safer integration profiles and less side-effects in a clinical setting, compared to currently used and improved versions of MLV and HIV-based vectors.

However, we think that integrating viral hybrid vectors will be beneficial for the treatment of some diseases originated from rapidly dividing cells like stem cells, keratinocytes or cancer cells. Within these large groups of target cells, hybrid vectors may find an important niche for transduction of those cell types for which MLV and HIV-based are not suited. Furthermore, conventionally used integrating vectors are limited by their packaging capacity. For example retroviral and lentiviral vectors have a limiting packaging capacity of 8–10 kb. Therefore, the significantly higher packaging capacity of HDAdV- and HSV-based hybrid vectors will be the only solution for diseases which require stable transduction of cells with large transgenes (> 10 kb).

## Figures and Tables

**Table 1.** State-of-the-art of hybrid vector technologies. The mentioned delivery systems are helper-dependent adenovirus (HDAdV), herpes simplex virus type 1 (HSV-1), retrovirus (RV), and integrase defective lentivirus (IDLV). Integration machineries used in combination with these delivery vehicles are Sleeping Beauty (SB) transposase, bacteriophage integrase PhiC31, retrotransposon, adeno-associated virus (AAV; AAV-ITR or AAV-Rep protein-mediated), retrovirus (RV; LTR-mediated or retrovirus integrase mediated), zinc-finger nuclease (ZFN), and DNA-binding domain-retroviral integrase-fusion proteins (DBD-IN fusion proteins). (+) represents the existing combination of the respective integration machineries and the delivering vector. (−) represents non-existing viral hybrid vector systems.

Integration machinery	SB	PhiC31	Retrotransposon	AAV	RV	ZFN	DBD-IN-fusion proteins
Delivering vehicle							
HDAdV	+	+	+	+	+	−	−
HSV-1	+	−	−	+	+	−	−
RV	−	−	−	−	/	−	+
IDLV	+	−	−	−	−	+	−

**Table 2.** Overview of currently used hybrid vector systems. The first column indicates the viral hybrid vector system for stable integration into the host genome. For each system the integration system and the integration pattern (random versus site-specific) are mentioned and the respective references are summarized. HDAdV: helper-dependent adenovirus, HSV-1: herpes simplex virus type 1, RV: retrovirus, IDLV: integrase defective lentivirus, SB: Sleeping Beauty transposase, PhiC31: bacteriophage-derived integrase PhiC31, AAV: adeno-associated virus, AAV-Rep: AAV-derived Rep coding sequence responsible for site-specific integration into AAVS1, ZFN: zinc-finger nuclease, DBD-IN fusion proteins: DNA-binding domain-retroviral integrase-fusion proteins.

Hybrid vector	Integrating system	Integration pattern	Reference
HDAdV/AAV	AAV-ITR	random	[38, 40–42]
HDAdV/AAVRep	AAV-Rep	site-specific	[30, 31, 43, 133]
HDAdV/SB	Sleeping Beauty transposase	random	[47]
HDAdV/PhiC31	PhiC31 integrase	randomly into *pseudo attP*-sites	[51]
HDAdV/retrotransposon	Retrotransposon	consensus genomic target DNA	[54, 55]
HDAdV/RV	Integrase	randomly into active genes	[27, 28]
HDAdV/FV	Integrase	random	[59]
HSV-1/SB	Sleeping Beauty transposase	random	[71]
HSV-1/AAVRep	AAV-Rep	site-specific	[65–70]
HSV-1/RV	Integrase	randomly into active genes	[74]
RV/IN	DBD-IN-fusion protein	site-specific	[9–97]
IDLV/ZFN	ZFN	site-specific	[101, 103]
IDLV/SB	Sleeping Beauty transposase	random	[105, 106]
